# Composite Membrane Electrodes Based on Graphite Materials from Lignin: Formation and Properties

**DOI:** 10.3390/polym18141706

**Published:** 2026-07-10

**Authors:** Mikhail Serbinovsky, Olga Popova

**Affiliations:** 1 Department of Life Safety, Rostov State Transport University, Rostovskogo Strelkovogo Polka Narodnogo Opolcheniya Sq. 2, Rostov on Don 344038, Russia; 2 Department of Life Safety and Environmental Protection, Don State Technical University, Gagarin Sq. 1, Rostov on Don 344000, Russia

**Keywords:** lignin-derived graphite materials, graphite bisulfate, thermally expanded graphite, paste composition, membrane formation, mechanical properties, thermogravimetric analysis, elemental analysis

## Abstract

The formation technology, structure, and properties of composite membranes based on graphite materials obtained from hydrolytic lignin are studied. Lignin, a large-tonnage polymer waste with high energy potential, is an environmentally friendly and promising material for electrodes. Graphite obtained from lignin is characterized by high purity and fine dispersion, which contributes to its increased efficiency in electrochemical systems. Methods for forming membrane electrodes using various binders and solvents have been developed and have improved the mechanical and rheological properties of the composites. The introduction of surfactants and stabilizers into the paste-like composition contributes to the increased strength and ductility of the resulting materials. The study results show that optimization of the liquid phase composition and the selection of suitable binders are key factors in achieving high-performance characteristics in electrode materials. In particular, the use of aqueous–alcoholic solutions and various surfactants significantly improves the wettability and ductility of the pastes, facilitating the electrode formation process. The heat resistance of the obtained membranes is at least 630–650 °C, which makes them promising for use in modern energy storage systems.

## 1. Introduction

There has recently been a significant increase in interest in alternative energy sources due to the shortage of fossil fuels. Therefore, the importance of renewable energy, including biomass, solar, and wind generators, is growing [1,2]. However, to ensure a stable energy supply, such sources must be combined with modern energy storage systems [1]. The development of new materials for use in electrochemical devices, including batteries, supercapacitors, and other modern energy storage systems, is of particular importance. Numerous studies are related to composite electrodes based on natural carbon from various sources, such as marine biomass [3], fruit waste [4], and others, for example [5,6,7]. Biocarbon is indispensable for the development of biodegradable battery systems characterized by low production costs and a low environmental footprint [8].

Lignin, a large-scale polymer waste product from the hydrolysis and pulp and paper industries, holds a unique place among natural carbon sources. Due to its low cost, high specific power and energy density, as well as its environmental friendliness and functionality, lignin is considered to be a promising electrode-active material [9]. It should also be noted that the change in the structure of lignin determines its new chemical properties [10], which is the basis for the development in the field of green-friendly bioelectronics [11]. Interest in the use of lignin has increased significantly in the last decade [12,13,14].

In modern energy storage technologies, batteries and supercapacitors are the most promising and important components [15]. Lithium-ion batteries are recognized as a key technology for portable electronics, power tools, and hybrid and fully electric vehicles, primarily due to their high energy and power density [16]. Battery cell components and design are constantly being improved to improve service life and stability, increase energy density and reduce cost [17]. An important aspect of further battery development is increasing the proportion of active material and reducing the proportion of inactive electrode components, such as binder and conductive additive [18]. Research to improve electrode chemical materials and manufacturing methods is crucial to achieving better electrochemical, microstructural, and mechanical performance, as well as battery economics and environmental sustainability [19,20].

Graphite is an ideal and strategically important electrode material for lithium-ion batteries and dominates the anode material family [21]. Interstitial compounds of graphite enable the production of low-density thermally expanded graphite (TEG) with a large specific surface area, which has promising applications as an electrode material [22,23,24] particularly in double-layer capacitors [25,26,27]. However, natural graphite contains foreign elements, which, even in low concentrations, can negatively impact the performance, stability, or safety of devices and processes [28], and methods for purifying them are not simple or inexpensive [29,30].

Graphite obtained from hydrolytic lignin (HL) has a graphitization degree of at least 94% and is characterized by high purity and fine dispersion. Its dispersion occurs directly during thermolysis and is independent of the milling degree of the feedstock [31]. This is due to the fact that a significant portion of lignin molecules consist of compounds with condensed benzene rings, and under conditions of oxygen deficiency, the active groups are split off without significant destruction of the aromatic system, which creates an optimal structure for the formation of graphite [30]. Previously, studies on the thermal and electrochemical modification of HL were presented in publications [31,32]. The patterns of change in the granulometric composition of HL during the sequential production of carbon material, graphite and graphite bisulfate (a graphite interstitial compound from HL (GB)) were established; the features of the structure and dispersed composition of TEG were studied and substantiated.

The high efficiency of using HL graphite as a matrix for producing graphite interstitial compounds and the effectiveness of TEG as a component of the active masses of lithium-ion battery electrodes were previously demonstrated in publications [33,34]. To conduct comprehensive studies to substantiate the use of lignin-derived graphite materials in batteries and supercapacitors, it is necessary to develop a technology for forming membrane electrodes.

In this paper, the component composition of blanks for membrane electrodes based on graphite materials from HL was developed, their strength and deformation characteristics were determined depending on the component composition, and the microstructure, elemental composition, thermal and electrochemical properties of composite electrodes were presented.

## 2. Materials and Methods

The objects of the study were graphite materials obtained on the basis of HL. Graphite was obtained in two stages. Carbon material was obtained by thermolysis of hydrolytic lignin of deciduous wood in a graphite container at a temperature of 600 °C without air access [31]. Then, the carbon material was graphitized at a temperature of 2800 °C for 1 h. The obtained graphite was dispersed in a 10 mol/L solution of H_2_SO_4_ (reagent grade) and GB was obtained in the process of electrochemical synthesis on a P-30I potentiostat-galvanostat (ELINS Ltd., Moscow, Russia) at a potential of ~1.5 V relative to a silver chloride electrode [35]. Stainless steel 12X18H10T was used to make the electrodes. The volume ratio of graphite: H_2_SO_4_ was 1:2. Next, GB was heated for 5–10 s at 750–900 °C to obtain TEG.

To determine the bulk density of TEG, a sample of the material was uniformly poured into a 100 mL cylinder, the powder mass was determined, and its density was calculated. The bulk density of the TEG samples was 2–2.5 g/dm^3^. The calculated specific surface area of TEG averaged 180 m^2^/g with a particle size of 10–50 µm and a bulk density of 2 g/dm^3^.

Pastes for forming electrode blanks were prepared by mixing the solvent and binder (15–20 wt%) and then adding GB to this solution. The following were used as binding components: Na-carboxymethyl cellulose (Na-CMC), polyvinyl acetate (PVA), PF-283 varnish (GOST 5470-75) (solutions of alkyd resins modified with vegetable oils, fatty acids of vegetable oils and products of their processing, fatty acids of tall oil and distilled tall oil in organic solvents with the addition of a siccative), nitrocellulose varnish NC-218 (GOST 4976-83) (colloidal solution of varnish collodion (nitrocellulose) in a mixture of solvents, alcohols and thinners with the addition of synthetic resins, plasticizers and special additives), and rubber glue NK based on natural rubber (GOST 2199-78).

The solvents used were distilled water or aqueous solutions of ethanol, butanol, propanol, isopropanol, acetone, white spirit, gasoline solvent (Nefras C2-80/120), and others. The compositions of GB-based pastes varied: GB—85–98 wt%, binder—2–15 wt% by dry matter. Carboxylic acids and carboxylic acid salts, sodium lauryl sulfate, emulsifier OP-7 (OP-10), polyvinyl alcohol (PVOH), and others were studied as surfactants.

The paste components were mixed in a mechanical mixer with a rotor speed of 60–100 rpm for 4–8 min. The viscosity characteristics of the pastes were assessed using an Anton Paar RheolabQC rotational rheometer (Anton Paar GmbH, Graz, Austria). A BM08/Q1 ball measuring system (8 mm ball diameter) was used. At a fixed shear rate of 0.3 s^−1^, the determined dynamic viscosity was in the range of 350–400 Pa·s.

Membrane blanks with thicknesses ranging from 0.5 to 5 mm were produced by single- or double-sided spreading or rolling a GB-based paste onto substrates consisting of the following:

– Metal woven and punched (stretched) steel meshes with a thickness of 0.1–1.5 mm with an opening coefficient of 0.38–0.92 (steel 20, 08Ch18H10T, 12Ch18N10T);
– Perforated metal tapes and plates made of NP2 nickel and iron (arm-co).

Paste formation by spreading or rolling was carried out using guide plates of a specified thickness as a stop for the spatula. Subsequent drying and heat treatment of the blanks at 750–900 °C were carried out between parallel limiting plates made of 12Ch18N10T steel, which ensured the required membrane thickness was maintained. The length of the finished membranes was 100 mm and the width was 40 mm.

Uniaxial tensile tests to determine tensile strength were performed at room temperature using an HUS-2010Z electrohydraulic tensile testing machine (Jinan Shijin Group Co., Ltd., Jinan, China). The loading rate was 10 mm/min. The tensile strength and residual elongations of the specimens were calculated using Equations (1) and (2).

(1) $$\;\sigma_{T}=\frac{F_{T}}{A},$$

Here, $\sigma_{T}$—tensile strength (Pa); $F_{T}$—test load (N); A—cross-sectional area of the specimen (N/m^2^ or MN/mm^2^).

(2) $$\varepsilon_{T}=\frac{{100(L}_{1}-L_{0})}{L_{0}}.$$

Here, $\varepsilon_{T}\;$—residual elongation (%); $L_{0}$—initial length of the specimen (mm); and $L_{1}$—length of the specimen before destruction (mm).

Thermogravimetric studies were conducted on a Diamond TG/DTA Perkin Elmer Q1500D thermogravimeter (derivatograph) (PerkinElmer, Waltham, MA, USA). Samples were heated at a rate of 10 °C min^−1^ to 1000 °C in air in platinum crucibles. The temperature measurement error was no more than 2%.

Electrochemical studies were conducted in a three-electrode cell at 25 °C using a P-30I potentiostat against a non-aqueous silver chloride reference electrode. Measurements were performed in an anhydrous 1 mol/L LiClO_4_ solution in a mixture of propylene carbonate and dimethoxyethane (1:1 vol). The electrode working surface area was 1 cm^2^, and spectral graphite served as the counter electrode. Before the experiment, the cell was subjected to standard treatment, including washing with a soda solution and bidistilled water, drying and a final rinse with a working electrolyte.

The diffusion-kinetic parameters of lithium intercalation were calculated using the Cottrell equation:

(3) $$K=\frac{\Delta i}{\Delta (1/\sqrt{t})}=\frac{zFC_{Li}^{0}\sqrt{D_{Li}}}{\sqrt{\pi }},$$

Here, i is the current density (A/cm^2^); z is the number of electrons in the electrode process; F is the Faraday constant, 96,485 (C/mol); $C_{Li}^{0}\;$is the initial lithium concentration (mol/cm); $D_{Li}$ is the lithium diffusion coefficient (cm/s); and t is time (s).

The microstructure and elemental composition of the samples were examined using a Quanta 200 scanning electron microscope (FEI Company, Hillsboro, OR, USA) equipped with an EDAX energy-dispersive X-ray spectroscopy (EDS/EDX) system.

The reliability of all measurements was ensured by repeating each test at least 3–5 times. The specified confidence interval was 10%.

## 3. Results and Discussion

A distinction is made between wet and dry methods of forming membrane electrodes [36,37], each of which has advantages and disadvantages depending on the implementation features and the materials used. In the wet method of electrode production, the use of aqueous solutions is considered optimal, since this approach improves the environmental friendliness of the technology and helps to reduce production costs [38,39]. Obtaining a uniform density of membranes based on TEG requires uniform distribution of the GB layer over the surface of the substrate (mesh or plate) or mold before foaming the GB. Experiments have shown that without a binder, it is possible to uniformly distribute GB only on a large-mesh mesh by filling its cells with particles; however, this method is not technologically advanced and requires an additional supporting surface [40,41]. In addition, the thickness of the workpiece is limited by the thickness of the mesh itself. A more technologically advanced method is to apply the paste (aqueous or non-aqueous) with a spatula or roller onto the substrate, followed by drying to remove the solvent and foaming. Pasting is a widely used membrane-forming process in which paste-like masses are applied to substrates using spatulas, blades, rollers, extrusion, or free flow of paste through dies, nozzles, holes, or slots in bins [42,43,44]. In industrial production, the paste application process is fully mechanized and automated, ensuring high productivity. This method is characterized by minimal mass loss and improved environmental friendliness compared to technologies using powders. Due to these advantages, the method is promising for the production of carbon electrodes for various purposes [45,46,47].

### 3.1. Development of Compositions of Squeous and Aqueous-Alcoholic Pastes Based on GB

**Figure 1:**
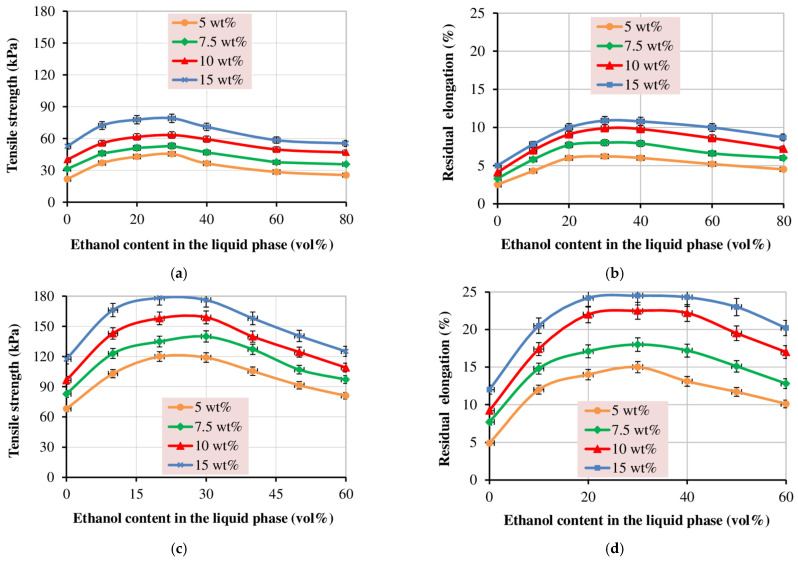
The effect of ethanol content in the liquid phase on the mechanical properties of unreinforced blanks based on GB with a binder content of 5–15 wt%. Binder: (**a**,**b**)—Na-CMC; (**c**,**d**)—PVA.

The study showed that replacing water with aqueous-alcoholic solutions in pastes improves the wettability of the components and stabilizes their rheological properties. The addition of ethanol ensures better wetting of the GB, increases paste plasticity, and facilitates its application with a spatula or roller. The strength and residual elongation of the composites reach their maximum values at an ethanol content of 15–35 vol% for composites with PVA and 25–35 vol% for composites with Na-CMC (Figure 1).

With a further increase in ethanol content, both parameters decrease, and at ethanol concentrations above 50–60 vol%, the plasticity of the pastes deteriorates sharply, i.e., the pastes cease to form a continuous layer when applied or molded with rollers. The need for a higher alcohol concentration in Na-CMC composites to achieve maximum strength and ductility suggests limited wetting of GB particles by the aqueous Na-CMC formulation. PVA-based composites exhibit higher strength and ductility (Figure 1c,d). Moreover, the performance of Na-CMC composites is also sufficient for technological operations: forming reinforced structures and filling volumetric or plate filters in equipment molds.

The strength and ductility of the composites increase almost proportionally to the increase in binder content (Figure 1). For composites with Na-CMC, a more significant relative increase is observed (1.7–2.4 times) than for composites with PVA (1.5–1.7 times). This is likely due to the presence of vinyl acetate in the PVA dispersion (up to 0.8% according to GOST 18992-80), as well as stabilizers (polyvinyl alcohol) and plasticizers (dibutyl phthalate). Vinyl acetate apparently improves the wettability of the GB particle surface by the binder and increases the strength of the composite. Polyvinyl alcohol acts as a plasticizing agent, increasing the ductility of the prepared composites.

**Figure 2:**
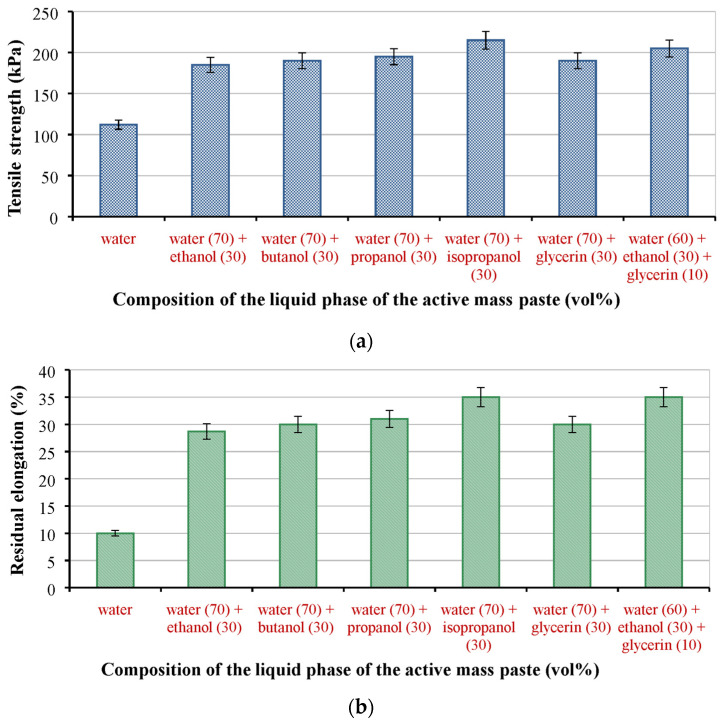
The influence of the composition of the liquid phase of the active mass containing 15 wt% PVA binder on the mechanical parameters of GB-based composites: (**a**) tensile strength; (**b**) residual elongation.

The use of aqueous-alcoholic solutions of butanol, propanol, and isopropanol also improves the wettability of GB by the liquid phase and increases the plasticity of the paste. Similar dependences of the strength and deformability of the composites are observed for these alcohols, as in the case of ethanol. However, the optimal ranges of alcohol content, at which maximum strength and ductility are achieved, differ. For composites with Na-CMC, this range is shifted toward lower alcohol content: for butanol it is 20–30 vol%, for propanol it is 18–30 vol%, and for isopropanol it is 15–30 vol%. In the case of composites with PVA, the maximum values for butanol and propanol are practically the same as those for ethanol (Figure 2).

When isopropanol is added to PVA-based composites, their strength increases as the alcohol concentration increases. However, the material’s ductility changes nonlinearly: it peaks at an isopropanol content of 15–30 vol%, and with further increases in isopropanol content, it decreases by 10–18% from this maximum. This decrease in ductility is explained by the solvent effect of isopropanol on PVA.

With high ethanol and butanol content in the liquid phase, the plasticity of the pastes decreases and their thixotropy increases, requiring the addition of stabilizers and surfactants. Adding glycerin at a concentration of 5–10 vol% to the liquid phase, along with the aforementioned alcohols, increases the plasticity and stability of the paste and reduces its thixotropy. It should be noted that the addition of propanol, isopropanol, and glycerin to the liquid phase increases the drying time of the pastes. Nevertheless, isopropanol and glycerin are recommended as components of the liquid phase of pastes.

It has been established that treatment of GB with an ethanol solution before the introduction of a water-soluble binder without subsequent drying increases the strength of composite blanks by 1.2–1.5 times, depending on the binder content.

When studying the influence of various surfactants and stabilizing additives on the mechanical characteristics of composites, additives were introduced into the liquid phase in the range of 0.1–1%. It has been proven that the studied surfactants improve the wettability of GB by the liquid phase and facilitate the formation of homogeneous composite layers. Thus, the paste is better applied to the substrate with a spatula or roller, and its thixotropy is reduced. In addition, the pot life of pastes increases, especially with a PVA binder. The introduction of a surfactant increases the strength and ductility of composite workpieces (Table 1).

The beneficial effect of surfactants is more pronounced in aqueous formulations than in aqueous-alcoholic ones. The best results in terms of composite strength and ductility were obtained by adding sodium lauryl sulfate to the liquid phase of the paste. However, the relative improvement compared to other surfactants is small, so the surfactant should be selected based on the principle of ensuring minimal impurities in the membranes after GB foaming and the lowest cost of the surfactant.

Adding surfactants and stabilizers to the paste significantly improves the rheological properties of the paste and maintains its plasticity for 4–8 h. Furthermore, during the molding process, i.e., as the paste is consumed, a new batch of paste can be added to the previous batch without compromising the quality of the molded blanks.

During the drying of composite blanks with Na-CMC and PVA binders, their strength increases as moisture is removed. However, at low moisture content, the plasticity of the blanks decreases, which adversely affects the integrity of the composites during subsequent processing steps. Therefore, pre-drying before foaming was carried out to a relative moisture content of 5–10%. Final moisture removal occurred during the final heat treatment of the membrane blanks.

### 3.2. Development of Non-Aqueous Paste Compositions Based on GB

The study examined the possibility of using non-aqueous solutions of binders to produce molding pastes. It was experimentally confirmed that pastes based on GB, industrial varnishes (specifically, nitrocellulose NC-218 and pentaphthalic PF-283), and rubber adhesive (NK) are suitable for forming composite blanks using spatula application and roller rolling methods. When using the roller molding method, it was found that, due to the high adhesion of the pastes to the working surfaces (both metal and polymer), the use of anti-adhesive coatings is mandatory for the successful completion of the process. A comparative analysis of the mechanical properties of the obtained materials showed that the strength and plasticity characteristics of composites based on NC-218 and PF-283 varnishes, as well as NK adhesive, are inferior to those of composites manufactured using a PVA dispersion. However, these same properties exceed those of composites using Na-CMC as a binder. The quantitative results of this comparison are presented in Table 2 (compared with the data in Table 1).

Drying of composites using organic-soluble binders before foaming the blanks was carried out for 40–50 min at room temperature or 10–15 min at 60–90 °C. For composites with NK adhesive, this process took 10–20 min at room temperature or 5–8 min at 40–60 °C. The paste pot life was 20–30 min in an open container and at least an hour in a closed container. To ensure continuous molding of the blanks, the paste was fed from a closed container through a die, allowing for the paste to be replenished in portions without degrading the blank quality.

Composites with NK adhesive have greater plasticity compared to other materials, allowing them to be molded into long, wide, or continuous strips less than 1 mm thick. These strips retain their flexibility even after the paste has dried and the solvent has been removed. This makes rubber-based binders promising for creating membranes and electrodes in the form of thin plates or long ribbons. However, their drawback is the use of flammable components and harmful organic solvents.

### 3.3. Study of the Structure, Elemental Composition and Thermal Stability of Composite Membranes

**Figure 3:**
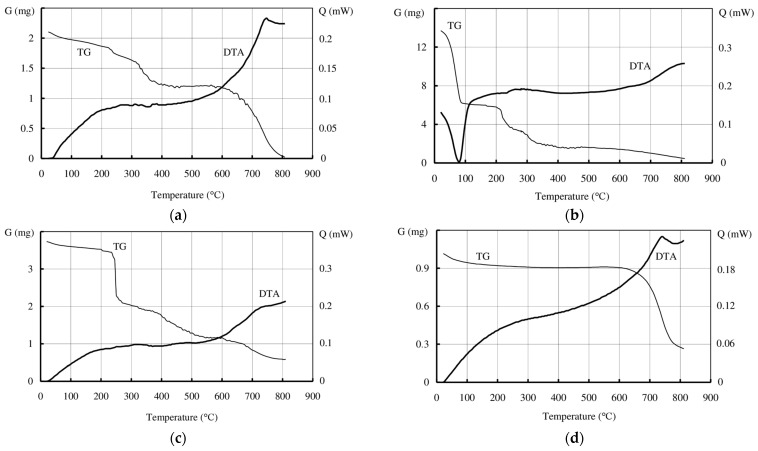
TGA curves: (**a**) GB; (**b**) GB paste with Na-CMC binder; (**c**) dry mixture of GB with Na-CMC binder; (**d**) membrane based on TEG.

Thermogravimetric analysis (TGA) results for the studied materials demonstrate high thermal stability in the low-temperature range. Specifically, the GB thermogram shows no noticeable changes up to 250 °C (Figure 3a). Upon further heating, deintercalation of sulfate ions apparently begins, intensifying at temperatures above 350 °C and continuing up to 450–470 °C. Oxidation of the expanded graphite is observed at temperatures above 650–700 °C, accompanied by significant mass loss and an exothermic effect.

When heating wet GB paste with Na-CMC, endothermic peaks are recorded on the thermogram at 40–110 °C and 220–250 °C (Figure 3b). The first peak is associated with the evaporation of free moisture; the second is associated with the removal of adsorbed water. This is followed by the processes of binder decomposition and oxidation, partial carbonization, and sulfate ion deintercalation, which are completed by 450–460 °C. Oxidation of soot particles (residues of carbonized binder) is observed at 600–610 °C, and graphite oxidation begins at 650–670 °C, continuing until 780–800 °C. GB foaming is not recorded in the presented curves, since its intensity is significantly lower than the processes of decomposition, oxidation, and deintercalation.

Thermogravimetric analysis of a dry GB powder with Na-CMC (Figure 3c) yielded results similar to those of a paste based on the same components. The main difference is the absence of effects related to moisture evaporation in the temperature range of 40–110 °C for the dry powder.

Heating the finished membrane, obtained by heat treating a dry GB preform with a binder at 750–900 °C, reveals no mass change or thermal effects up to 670–680 °C (Figure 3d). At 680–780 °C graphite oxidation begins, accompanied by mass loss and an exothermic effect. Consequently, the thermal stability of the membranes studied is at least 630–650 °C.

**Figure 4:**
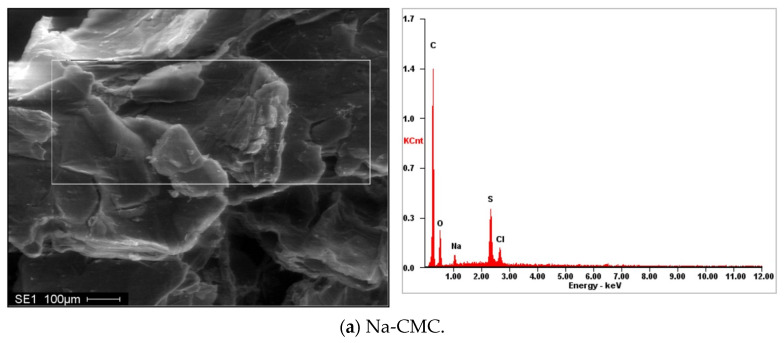

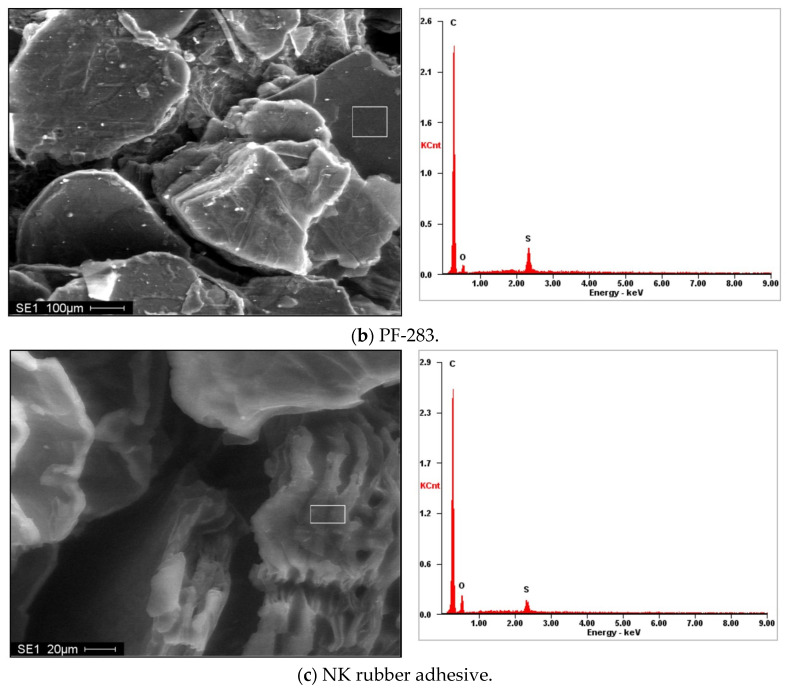
Microstructure and elemental analysis results of GB-based pastes with different binders.

Elemental analysis of microzones of graphite bisulfate composite blanks with a Na-CMC binder revealed the presence of sulfur in the samples (Figure 4a). After heat treatment (foaming) during membrane formation, sulfur remains, indicating that the 750–770 °C temperature regime is insufficient to completely remove sulfur and metal compounds from thermally expanded graphite. When the processing temperature is increased to 850–900 °C, sulfur is not detected in the samples; however, traces of sodium, calcium, and copper are detected. Their presence is explained by contamination of the original industrially produced hydrolytic lignin, as well as the presence of these elements in surfactants and stabilizers included in graphite bisulfate-based binding pastes. Therefore, metal- and sulfur-free components should be used for the manufacture of membrane electrodes.

A graphite bisulfate-based composite using PF-283 binder (Figure 4b) is characterized by high chemical purity and contains only three elements: carbon, oxygen, and sulfur. A similar level of purity is observed in composites using rubber adhesive as a binder (Figure 4c).

Analysis of the lateral surface of the particles where the graphite planes are exposed shows that the sulfur and oxygen contents here are approximately three times higher (17.65 and 3.80 wt%, respectively) compared to a particle analysis in transmission mode. This indicates that sulfate ions are localized primarily in the interplanar spaces of the graphite. Binders based on Na-CMC and PF-283 form continuous films on the surface of graphite bisulfate particles. At the same time, binders based on rubber adhesive and polyvinyl acetate form visually noticeable “bridges” between the graphite particles. It is probably due to this structure that composites with these binders have higher plasticity.

**Figure 5:**
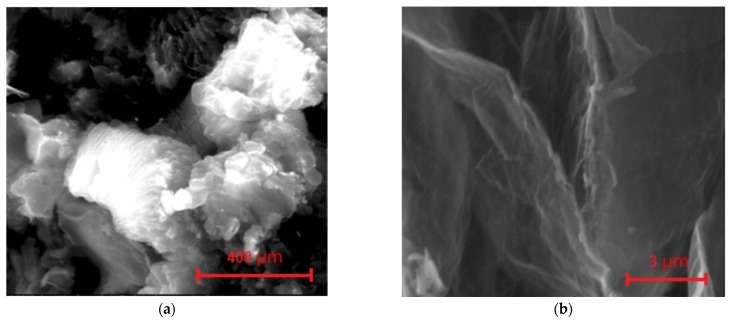
Microstructure of a membrane based on TEG: (**a**)—200×; (**b**)—20,000×.

TEG-based membranes consist of interwoven lamellar particles that, when foamed, form a unified structure, ensuring the integrity and mechanical strength of the material (Figure 5).

Due to their significant pore volume and lamellar particle morphology, these composites have a high specific surface area, which may be advantageous for future electrode applications.

### 3.4. Study of Composite Membranes as Electrode Materials

**Figure 6:**
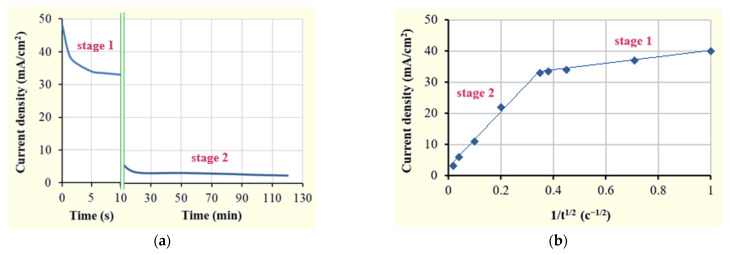
(**a**) Potentiostatic curve of lithium intercalation into a membrane electrode (E = −3.1 V, *t* = 2 h, T = 25 °C); (**b**) dependence *i* − 1/*t*^1/2^ for calculating kinetic parameters.

As part of the electrochemical studies of composite membranes, the kinetics of lithium intercalation into the graphite matrix was examined, followed by an analysis of the discharge characteristics of the LiXC_6_ electrode. Electrochemical intercalation was conducted in potentiostatic mode at a potential of −3.1 V for 2 h. According to an analysis of the time dependence of the current density (Figure 6a), the process begins in the range of 48–35 mA/cm^2^ and stabilizes at 3–4 mA/cm^2^ after 10 min.

To identify the kinetic regularities of the process, the *i* − *t* dependence was transformed into *i* − 1/*t*^1/2^ coordinates (Figure 6b). The break in the linear dependence indicates a two-stage process and is consistent with the data obtained previously in [48,49]. In the first stage, a passivating film is formed on the graphite surface with conductivity via Li ions, and in the second stage, Li is introduced into the deep layers of graphite with the formation of Li_X_C_6_ compounds.

The calculated data on the kinetic characteristics for electrodes manufactured for comparative measurements based on crucible graphite and low-ash graphite according to publication [34] are also given in Table 3. The rates of lithium intercalation into the graphite structure (stage 2) are comparable for the materials studied. However, the maximum rate of surface film formation (stage 1), observed for GT, indicates the formation of a denser layer, which subsequently has a negative impact on the electrode’s charge–discharge characteristics.

**Figure 7:**
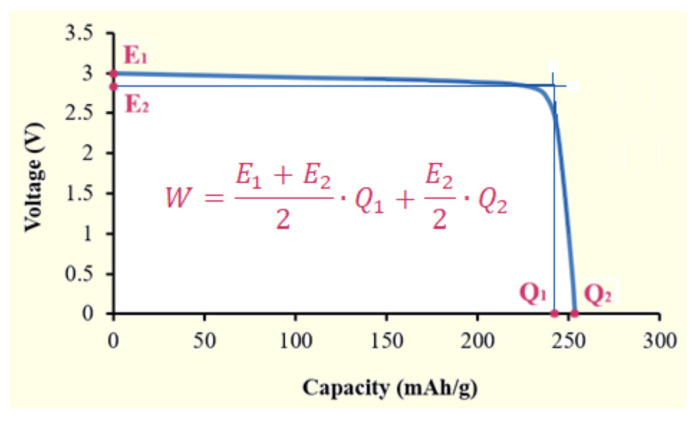
Discharge curve of Li_X_C_6_ composite membrane electrode (*i* = 0.03 mA/cm^2^, T = 25 °C).

During the galvanostatic discharge of Li_X_C_6_ electrodes at a current density of 2 mA/g (0.03 mA/cm^2^), anodic chronopotentiograms were obtained (Figure 7), the analysis of which made it possible to estimate the specific capacity of the electrodes.

Composite electrodes based on graphite materials obtained from lignin demonstrate excellent energy capacity in comparison with analogs based on other carbon matrices. This effect is due to the homogeneous finely dispersed structure of thermally expanded graphite (TEG) from lignin, 65% of which consists of particles smaller than 50 μm. The high degree of foaming and opening of the graphite plates ensures an effective contact area [31]. Thus, graphite materials based on lignin represent a promising matrix for the manufacture of electrodes for lithium-ion batteries with high specific energy.

## 4. Conclusions

The research was conducted on the formation of membrane electrodes from TG by foaming with GB on mesh substrates under conditions of limited foaming volume upon heating the membrane electrode blanks to a temperature of 750–900 °C. The mechanical properties of membrane electrode blanks obtained by spreading and rolling pastes containing GB and a binder (Na-CMC, PVA, nitrocellulose varnish NC-218, pentaphthalic varnish PF-283, rubber adhesive NK) onto the substrates were investigated. Pre-treatment of GB with ethanol without drying before the introduction of a water-soluble binder was shown to be an effective way to increase the material’s strength by 1.2–1.5 times. The experimental dependences of the mechanical properties of composite blanks based on GB and binders (Na-CMC, PVA) on the concentration of alcohols (ethanol, propanol, isopropanol, butanol) in the liquid phase were found to be extreme. It was shown that the position of the maximum on the curves is determined by both the nature of the alcohol and the type of polymer binder.

The studies found that the addition of surfactants to the pastes improves their processing and mechanical properties, particularly strength, ductility, and plasticity retention time (4–8 h). Since the effect of various surfactants on the final strength of the blanks is insignificant, their selection should be based on criteria such as minimizing residual impurities after heat treatment and cost. The choice of binder, in turn, is determined by the requirements for the thickness and strength of the blank, as well as the membrane design.

Based on the data obtained, NK rubber adhesive and PVA are recommended as binders for the fabrication of TEG-based membrane electrodes. Their use ensures a combination of high purity of the composite membranes and adequate mechanical properties of the preforms.

Thermogravimetric analysis (TGA) data for TEG-based membrane samples from HL indicate their high thermal stability, confirming the material’s thermodynamic stability over a wide temperature range, from room temperature to at least 630–650 °C.

Electrochemical tests of membrane electrodes based on TEG synthesized from HL were conducted, compared with electrodes based on TEG made from crucible graphite and special graphite with low ash content. The obtained data demonstrate the potential of a graphite matrix obtained from lignin for the creation of lithium-ion battery electrodes that provide high specific energy.

## Figures and Tables

**Table 1:** Tensile strength and residual elongation of composite blanks based on GB with Na-CMC and PVA binders when molded from pastes with a liquid phase of different compositions.

Binder content (wt%)	**Composition of the binder (vol%)**							
	water	water + ethanol 30%	water + oleic acid 1%	water + ethanol 29% + oleic acid 1%	water + sodium lauryl sulfate 1%	water + ethanol 29% + sodium lauryl sulfate 1%	water + OP-7	water + ethanol 29% + OP-7 1%
	**Tensile strength of composite blanks with Na-CMC binder (kPa)**							
5	21.8	45.5	29.0	52.0	32.8	55.0	28.0	53.0
7.5	31.3	53.1	41.0	61.0	44.0	65.0	42.0	58.5
10	40.0	63.3	53.0	72.0	54.9	76.7	51.0	68.3
15	52.7	79.0	70.0	87.2	74.5	90.5	71.0	84.5
	**Residual elongation of composite blanks with Na-CMC binder (%)**							
5	2.5	6.2	3.3	7.3	3.6	8.2	3.5	8.0
7.5	3.4	8.0	4.4	9.4	5.0	10.2	4.7	10.1
10	4.2	9.8	5.4	11.6	6.0	12.5	5.9	12.3
15	5.1	10.9	6.6	12.8	7.3	13.9	7.1	13.7
	**Tensile strength of composite blanks with PVA binder (kPa)**							
5	68.0	119.0	79.7	125.0	89.0	132.0	84.3	127.0
7.5	82.8	140.0	95.8	142.9	104.0	149.0	99.9	147.0
10	96.4	159.0	106.8	161.2	113.9	167.2	111.1	165.0
15	117.4	176.2	134.6	185.2	133.0	190.0	136.3	182.5
	**Residual elongation of composite blanks with PVA binder (%)**							
5	4.7	15.0	6.1	18.5	6.0	18.1	6.8	19.2
7.5	7.6	18.5	9.9	22.8	10.2	22.0	10.2	24.0
10	9.5	22.7	12.1	27.8	12.0	27.1	12.7	29.2
15	11.4	24.6	14.5	29.9	14.2	28.9	14.9	30.9

**Table 2:** Tensile strength and residual elongation of composite blanks based on GB and organosoluble binders.

Binder content, wt%	**Composition of the binder (vol%)**		
	Nitrocellulose varnish NC-218	Pentaphthalic varnish PF-283	Rubber adhesive NK
	**Tensile strength of composite blanks (kPa)**		
5	50.4	61.1	63.0
7.5	66.2	83.0	91.0
10	73.2	93.8	115.7
15	89.6	111.5	132.9
	**Residual elongation of composite blanks (%)**		
5	4.4	8.7	24.3
7.5	5.6	9.3	26.8
10	7.6	9.7	32.4
15	8.7	11.2	43.2

**Table 3:** Diffusion-kinetic parameters of lithium intercalation into composite graphite electrodes.

Source Materials for TEG	K∙10^3^ (A∙c^1/2^∙cm^−^^2^)		C^0^_Li_√D_Li_∙10^7^ (mol∙cm^−^^2^∙c^−^^1/2^)		W (W h/kg)
	Stage 1	Stage 2	Stage 1	Stage 2	
Graphite obtained from HL	11	98	2.0	17.6	720–750
Crucible graphite (GT-1) [34]	31	120	5.7	22.0	210
Special graphite with low ash content (GSM-1) [34]	3	117	0.6	21.0	170

## Data Availability

The original results presented in this study are included in the article. Additional questions can be directed to the authors.

## Abbreviations

The following abbreviations are used in this manuscript:

HL	Hydrolytic lignin
GB	Graphite bisulfate
TEG	Thermally expanded graphite
Na-CMC	Na-carboxymethyl cellulose
PVA	Polyvinyl acetate
PVOH	Polyvinyl alcohol
NC-218	Nitrocellulose varnish
PF-283	Pentaphthalic varnish
NK	Rubber adhesive
TGA	Thermogravimetric analysis
